# Non-Native (Exotic) Snake Envenomations in the U.S., 2005–2011

**DOI:** 10.3390/toxins6102899

**Published:** 2014-09-29

**Authors:** Brandon J. Warrick, Leslie V. Boyer, Steven A. Seifert

**Affiliations:** 1 NM Poison & Drug Information Center, MSC09 5080, University of New Mexico, Albuquerque, NM 87131-0001, USA; E-Mail: brandon_warrick@hotmail.com; 2 VIPER Institute, 1501 N. Campbell Avenue, Room 6131, Tucson, AZ 85724, USA; E-Mail: boyer@viper.arizona.edu

**Keywords:** exotic, non-native, envenomation, snakebite, Untied States, Poison Center, viper, elapid, antivenom, demographics

## Abstract

Non-native (exotic) snakes are a problematic source of envenomation worldwide. This manuscript describes the current demographics, outcomes and challenges of non-native snakebites in the United States (U.S.). We performed a retrospective case series of the National Poison Data System (NPDS) database between 2005 and 2011. There were 258 human exposures involving at least 61 unique exotic venomous species (average = 37 per year; range = 33–40). Males comprised 79% and females 21%. The average age was 33 years with 16% less than 20 years old. 70% of bites occurred in a private residence and 86% were treated at a healthcare facility. 35% of cases received antivenom and 10% were given antibiotics. This study is compared to our previous study (1994–2004) in which there was a substantial coding error rate. Software modifications significantly reduced coding errors. Identification and acquisition of appropriate antivenoms pose a number of logistical difficulties in the management of these envenomations. In the U.S., poison centers have valuable systems and clinical roles in the provision of expert consultation and in the management of these cases.

## 1. Introduction

In the United States (U.S.) 18% of homes have snakes, and $264 million is spent annually on pet snake(s) [1]. The importation of reptiles has declined dramatically as breeding and snake husbandry has taken hold in the U.S. In fact, the U.S. is currently the major exporter of snakes in the world [1]. There are institutional collections in zoos, aquariums and universities [2,3] and also a large private trade in venomous snakes [4,5]. As a result, there have been periodic reports of non-native envenomations in the U.S. [6,7,8,9]. The problem is not confined to the U.S., with reports of non-native envenomations occurring around the world [10,11,12].

We previously systematically described envenomations of non-native snake bites in the U.S. using the national poison center database (then called the Toxic Exposure Surveillance System, TESS, now called the National Poison Data System, NPDS) between 1994 and 2004 [13]. In that study, there were between 31 and 52 reports of envenomations by non-native snakes annually reported to U.S. poison centers. Over 70 different snake species were involved and pediatric exposures were found in similar proportions to native snakebites, suggesting at-risk household members. There was also a significant miscoding rate, with 60% of the cases initially coded as non-native bites actually being native snakes as well as a number of case duplications, where multiple poison centers submitted the same case to the database. As a result of that study, changes were made in to the coding system (POISINDEX) to more explicitly indicate which codes and snake names were for native and non-native snakes and revised the order of codes to display native snakes first. In addition, feedback was provided to centers to improve coding accuracy [14]. In the current study, our goals are to describe the current demographics of non-native snakebites and the effects of software modifications and poison center staff education on the rates of coding errors.

## 2. Methods

Cases coded as non-U.S. native venomous snake exposures reported to U.S. Poison Control Centers (PCC) and entered into the (NPDS) database between 1 January 2005 and 31 December 2011 were reviewed. Cases involving questionable snake identifications, those with similar common or Latin names, and suspected case duplications were confirmed with the reporting poison center. Cases of native snakebite apparently erroneously coded as exotics and case duplications were removed from the database before analysis. Viperid and elapid snake families were analyzed for differences in victim demographics, symptoms, signs, managements, and outcomes. There were insufficient numbers of hydrophid snakes for separate statistical analysis and these snakes were included in descriptive analyses only. Coding error comparisons were made before and after changes in POISINDEX in 2008. Statistical analysis including percent, means and confidence intervals was done by 2-tailed Fishers exact or Chi-squared test and performed using GraphPad InStat for Windows, Version 3.36. This work was granted an exemption from the Institutional Review Board review by the University of New Mexico.

## 3. Results

### 3.1. Confirmed Reports/Miscoding

**Figure 1:**
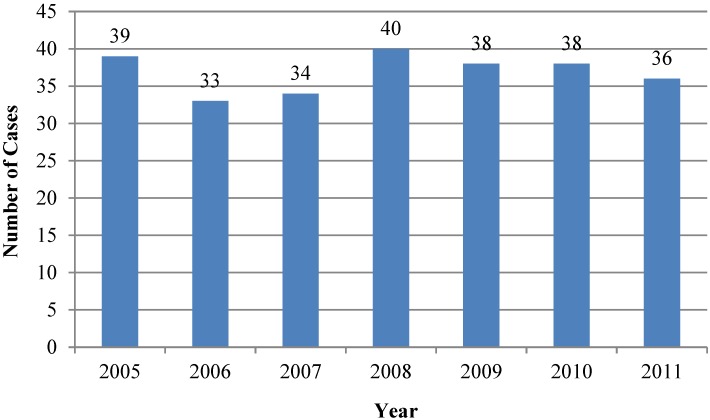
Confirmed non-native snake exposures, 2005–2011.

Between 1 January 2005 and 31 December 2011, there were 439 cases of non-native envenomations coded by U.S. Poison Centers. Of these, 258 were confirmed as caused by non-native snakes (average = 37 per year; range = 33–40) (Figure 1). Of the 181 miscoded cases, 178 reports involved native snakes miscoded as non-native species, including 169 native copperheads miscoded as *Austrelaps superbus*, *Denisonia superba* or *Deinagkistrodon acutus* and 9 cases of native rattlesnakes miscoded as non-native *Crotalus* species. There were 3 case record duplications, in which two centers both coded a case as the primary center (2) and one in which a center created 2 records of the same case.

The coding error rate for the years 2005–2008 was 56% and from 2009 to 2011, 14% (*p* < 0.0001). There were no cases of true *Austrelaps superbus*, *Denisonia superba*, or *Deinagkistrodon acutus* envenomations confirmed during this time period.

### 3.2. Types of Snakes Involved

Most snakes were identified by genus and species. Some snakes were reported by region (“African”; “Asian”; “Australian”; “European”; Middle Eastern”). In some of those cases, family identification was possible. By family, there were 39% elapidae, 43% viperidae, 2% hydrophiidae, and 16 undetermined. There were at least 27 different genera and 61 different species represented (Table 1).

**Table 1:** Non-native snake exposures reported to U.S. Poison Centers, 2005–2011.

Snake Genus and Species by Family	N
**ELAPIDAE**	
*Acanthophis antarticus*	1
*African snakes-Elapidae*	6
*Asian snakes-Elapidae*	5
Australian snakes	2
*Demansia olivacea*	2
*Dendroaspis angusticeps*	6
*Dendroaspis jamesoni*	1
*Dendroaspis polylepis*	11
*Dendroaspis viridis*	1
*Hemachatus haemachatus*	3
*Leptomicrurus narducci*	1
*Naja atra*	1
*Naja haje*	2
*Naja melanoleuca*	3
*Naja naja*	7
*Naja naja arabicus*	2
*Naja naja kaouthia*	17
*Naja naja sputatrix*	4
*Naja nigricollis*	14
*Naja pallida*	1
*Ophiophagus hannah*	6
*Oxyuranus scutellatus*	1
*Pseudonaja guttata*	1
*Pseudonaja nuchalis*	1
Spitting cobra, Asian	1
**HYDROPHIIDAE**	
*Aipysurus apraefrontalis*	2
*Ephalophis greyi*	1
*Hydrophis caerulesceus*	1
*Pelamis platurus*	2
**VIPERIDAE**	
*Agkistrodon bilineatus*	2
*Agkistrodon halys*	1
*Atheris chlorechis*	1
*Atheris squamiger*	2
*Bitis arietans*	2
*Bitis caudalis*	3
*Bitis gabonica*	10
*Bitis nasicornis*	2
*Bitis parviocula*	1
*Bothrops alternatus*	3
*Bothrops asper*	3
*Bothrops atrox*	4
*Bothrops godmanni*	4
*Bothrops lanceolatus*	6
*Bothrops lansbergii*	1
*Bothrops lateralis*	2
*Bothrops nasuta*	1
*Bothrops neuweidi*	1
*Bothrops nigroviridis*	2
*Bothrops ophroyomegas*	8
*Bothrops picadoi*	1
*Bothrops schlegeli*	3
*Boulengerina annulata*	1
*Bungarus magnimaculatus*	2
*Cerastes cerastes*	2
*Crotalus basiliscus*	2
*Crotalus durissus*	4
*Crotalus durissus terrificus*	2
*Crotalus unicolor*	1
*Deinagkistrodon acutus*	9
*Echis leuogaster*	1
European snakes	2
*Lachesis mutus*	9
*Lachesis mutus stenophrys*	3
*Sistrurus ravus*	2
*Trimeresurus albolabris*	1
*Trimeresurus elegans*	1
*Trimeresurus flavoviridis*	1
*Trimeresurus popeiorum*	1
*Trimeresurus stejnegeri*	2
*Vipera ammodytes*	2
*Vipera russelii*	1
**CANNOT BE CLASSED BY FAMILY**	
Middle Eastern snakes	24
**UNKNOWN**	16
**TOTAL**	258

### 3.3. Demographics of the Exposed Individuals

Seventy-nine percent of patients were males, 21% females. The average age was 33 years (range 16 months–87 years). Nine (4%) were less than 6 years of age; 7 (3%) were between 6 and 12 years; 21 (10%) were between 13 and 19 years; and 175 (84%) were aged 20 years or older.

### 3.4. Circumstances of the Exposure

**Figure 2:**
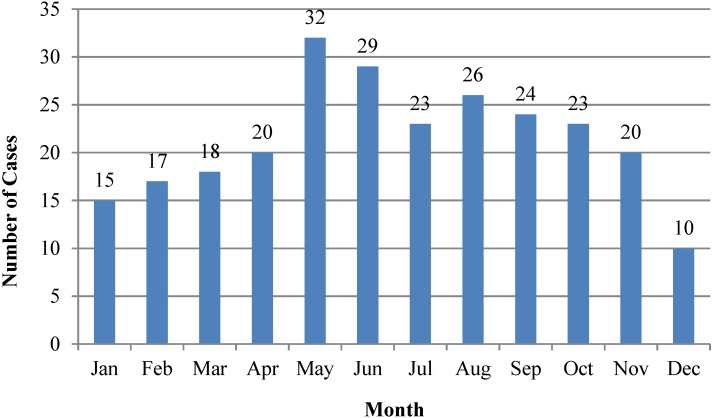
Non-native envenomations by month, 2005–2011.

Bites occurred more frequently between May and August (Figure 2). Seventy percent occurred in a private residence, 11% occurred at a workplace, 10% occurred in a public area and 9% occurred in another or unknown location. Ninety-six percent were coded as unintentional, 2% as intentional and 1 case as a malicious envenomation. Poison centers were initially contacted from a residence in 27%, from a healthcare facility in 58%, and from another or unknown location in 15%.

### 3.5. Management

Of the known management locations, 86% were managed at a healthcare facility and 9% were managed outside of a healthcare facility. Antivenom administration was documented in 35% of cases, and antibiotics were documented in 10% of cases. There were six cases (2.3%) coded with an adverse reaction to treatment.

### 3.6. Medical Outcomes and Duration of Clinical Effects

**Figure 3:**
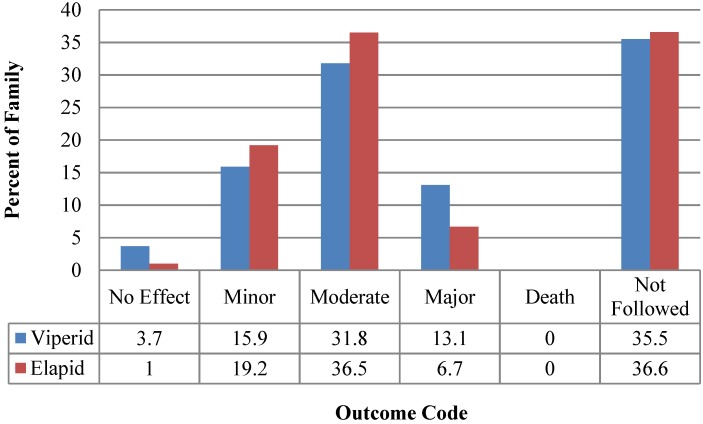
Viperid and elapid envenomation outcomes, 2005–2011.

**Figure 4:**
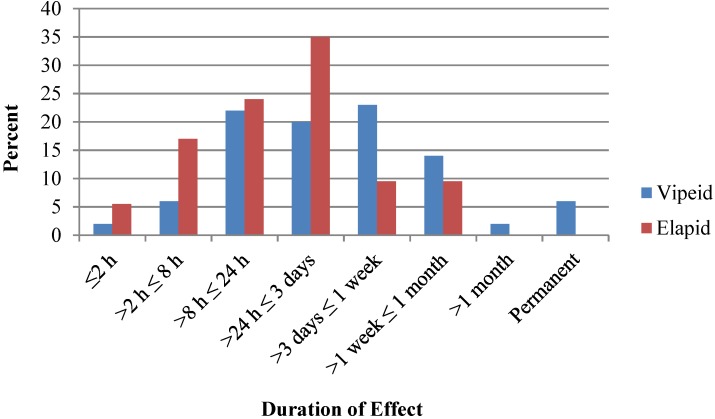
Viperid and elapid clinical effects duration, 2005–2011.

A comparison of known medical outcomes and effects duration comparing viperids and elapids are summarized in Figure 3 and Figure 4. In the five hydrophid cases, where outcomes and duration of effects were known, there were two minor, one moderate and one major outcome cases and two effect durations of between 3 days and one week. NPDS definitions of Medical Outcome and clinical effect codes are summarized in Appendix A. Thirty-six percent of cases were not followed to outcome.

## 4. Discussion

### 4.1. Demographics

In this follow-up study of non-native envenomations reported to U.S. poison centers, we note that the demographics of the victims of exposure have remained essentially the same and that the diversity of genera and species involved remains large, requiring numerous different, foreign (and non-FDA approved) antivenoms for definitive management. Once again, the incidence of pediatric exposures mirrors that seen in native envenomations, indicating a risk to household members of private collectors of venomous snakes.

### 4.2. Coding Errors

The incidence of coding errors, particularly in the miscoding of native envenomations as being from non-native species, was significantly reduced, particularly between 2009 and 2011 [13]. This appears to be primarily from changes made in the coding software, as feedback from the initial study appears to not have had much effect on coding errors. In the current, study the primary cause of continued coding errors was the continued use of the code for *Deinagkistrodon* (an Asian viper) for native *Agkistrodon* (U.S. copperhead or cottonmouth) envenomations, and the use of non-native *Crotalus* species codes for native rattlesnake envenomations. The remaining coding errors are from case duplications resulting from multiple centers being contacted simultaneously and independently. New administrative modifications are being implemented in POISINDEX and in data review in NPDS in an effort to reduce these remaining sources of coding error [14].

### 4.3. Non-Native Antivenoms/Adverse Reactions

Ideally, treatment of an exotic snake envenomation consists of timely administration of antivenom specific to the type of snake involved. Rates of antivenom use in NPDS are coded. However, the NPDS database does not indicate the type of antivenom given or other details of its administration. For example, we do not know what antivenoms were obtained, from where, how long that process took, or whether antivenoms were in-date or expired. Because of variable coding rates and non-specificity of clinical effect codes, we do not have an accurate or complete picture of adverse events and whether these were adverse drug reactions (ADR) to antivenoms or to other treatments. The documented ADR rate of 2.3% is well below reported rates of adverse reactions to antivenoms of between 5% and 75% [15,16].

There are many challenges to the health care system and to the individual practitioner in the management of these cases, making location, acquisition and administration of specific antivenom difficult, and sometimes impossible to accomplish. Unless they participate in Investigational New Drug Applications (INDAs), healthcare facilities are restricted to stocking antivenoms approved for marketing in the United States, and these are primarily effective in treating envenomations by North American creatures. Since the importation of foreign antivenoms into the US requires an INDA, among other requirements and impediments, private collectors are also highly unlikely to have antivenom for their snakes.

The current system for determining the appropriate antivenom for a particular snake species, locating an adequate amount of in-date product, and arranging for its transportation to the patient’s location, is based on zoo supplies of foreign antivenoms. Zoos obtain non-native antivenoms under a special INDA in case their workers are accidentally envenomated. As such, zoos only obtain antivenoms against the snakes in their collections. The national distribution of specific antivenoms is variable at best and may not include needed antivenom at all if a particular venomous snake is not included in any zoo collections. An online Antivenom Index allows zoos to list their antivenoms and contact information, for poison centers to establish contact between zoos and treating physicians and to provide information and consultative resources in the management of these cases [17]. Typically, the process of locating and transporting antivenom takes many hours, as the source of a specific antivenom may be geographically distant from the envenomated patient. Other challenges in this system include antivenom storage conditions and documentation, the use of unfamiliar biologic agents by hospital personnel, provision of clinical expertise, the possible use of expired product, reimbursement to the zoos, and local and federal regulatory compliance. The relatively low rate of antivenom use documented in the current study may reflect these barriers to antivenom acquisition and use.

### 4.4. Similar Problems around the World

The U.S. experience with non-native envenomations is similar to that elsewhere in the world. There were 404 exotic bites reported over an 11-year period in France and Germany [10]. In the United Kingdom, a report by four national poison information centers identified 510 cases of snake envenomation over a seven-year period, 133 (26%) involving non-native species [11]. Another report described 34 non-native envenomations over a 15-year period in the Czech Republic involving 31 different venomous snake species [12]. The demographics of envenomation victims reported in European reviews of non-native venomous reptiles are quite similar to our report and similarly large spectrums of non-native species are also commonly reported [11,12,18]. Reports of non-native envenomations elsewhere in the world also document difficulties with clinical unfamiliarity and challenges in obtaining appropriate antivenoms. In at least one report, it took 5 days to obtain an antivenom [12] and a green mamba (*Dendroaspis viridis*) envenomation in France also demonstrated logistical difficulties in obtaining antivenom in a timely manner [19]. In response, a centralized antivenom bank was developed by two regional poison centers in order to be able to deliver critical non-native antivenoms anywhere in France. These centers stock antivenom effective against 30 different types of venomous snakes [20,21]. A similar national antivenom depot has been operational in the Netherlands since 2008 [22].

### 4.5. Poison Center Training and Response

Policies and procedures should be in place at zoos and universities and other institutions that keep venomous animals to minimize the risk of envenomation. When an envenomation can be reasonably anticipated in a geographical location (e.g., when there are known zoo or other collections), simulated drills involving the animal facility and responding healthcare entities may improve response times and outcomes [23].

Since clandestine collections may exist anywhere, in the U.S., regional poison centers should be prepared to respond to exotic envenomations. Training of poison center staff should include the management of exotic envenomations, including the use of the online Antivenom Index.

## 5. Limitations

The NPDS database relies on passive reporting. It is unknown how many cases of non-native envenomation were not reported to Poison Centers. Underreporting may be more likely with dry bites and individuals who are in violation of exotic animal laws.

The rate of documentation of symptoms, signs, and managements appears to be low and variable. The low rate of adverse reactions to antivenom documented in this database, for example, is likely a significant under-documentation of this occurrence. Dramatic findings or interventions, such as intubation, may be more likely to be documented than something more mundane such as nausea or diaphoresis. Snake genus and species identification is often provided by the snake owner and may not be accurate. Effect durations may also reflect some imprecision as a significant number of cases were not followed to completion.

Retrospective studies may have patient selection bias, non-standardized patient assessment and management, and variability of data acquisition and documentation. More than one-third of these cases were lost to follow-up, limiting ultimate outcome information. Finally, conclusions regarding treatment efficacy cannot be drawn.

## 6. Conclusions

Non-native snake envenomations are reported to US Poison Centers between 33 and 50 times per year and most frequently involve private collectors. Zoos and other collections can prepare for the eventuality of an envenomation. However, since the largest proportion of envenomations is in private collections, and commonly remote from appropriate antivenom, a national system to manage these cases, is required. Zoo antivenom supplies, the online Antivenom Index, and the special expertise of regional poison centers provide the basic structure of the current U.S. system.

Changes in NPDS coding procedures appear to be effective at decreasing miscoding of native species as exotic envenomations in the NPDS database. Better data collection will allow better demographic description of these cases.

In addition, non-native snakebite and other envenomations are a global problem. Health system response is often characterized by confusion, inadequate preparations, and many challenges in management. In the U.S., poison centers have valuable systems and clinical roles in the provision of expert consultation and in the management of these cases.

## Appendix A: NPDS Category Definitions of Reasons for Exposure, Outcomes and Duration of Clinical Effects [24]

### A.1. Reason for Exposure

- Unintentional/general. An unintentional exposure resulting from an unforeseen or unplanned event.
- Intentional/suicide. An exposure resulting from the inappropriate use of a substance for self-destructive or manipulative reasons.
- Intentionality unknown. The reason for the exposure cannot be determined.
- Occupational. Any exposure that occurs as a direct result of the person being on the job or in the workplace.
- Adverse reaction. Adverse reactions to a product, including allergic, hypersensitive, or idiosyncratic response to a drug.

### A.2. Medical Outcomes

- No effect. The patient developed no symptoms as a result of the exposure.
- Minor. The patient exhibited some symptoms as a result of the exposure, but they were minimally bothersome to the patient.
- Moderate. The patient exhibited symptoms as a result of the exposure which are more pronounced, more prolonged or more of a systemic nature than minor symptoms.
- Major. The patient has exhibited symptoms as a result of the exposure which were life-threatening or resulted in significant residual disability or disfigurement.
- Death. The patient died as a result of the exposure or as a direct complication of the exposure where the complication was unlikely to have occurred had the toxic exposure not preceded the complication.

### A.3. Duration of Clinical Effects

The duration of clinical effect is defined as the time to resolution of all related clinical effects except those which are trivial or inconsequential. The following intervals are allowed:

- <2 h
- >2 h and ≤8 h
- >8 h and ≤24 h
- >24 h and ≤3 days
- >3 days and ≤1 week
- >1 week and ≤1 month
- >1 month
- Anticipated permanent
- Unknown

## Appendix B: Disclosure Statement on AAPCC Data

The American Association of Poison Control Centers (AAPCC) maintains the national database of information logged by the country’s 55 poison centers (PCs). Case records in this database are from self-reported calls: they reflect only information provided when the public or healthcare professionals report an actual or potential exposure to a substance (e.g., an ingestion, inhalation, or topical exposure, *etc.*), or request information/educational materials. Exposures do not necessarily represent a poisoning or overdose. The AAPCC is not able to completely verify the accuracy of every report made to member centers. Additional exposures may go unreported to PCs and data referenced from the AAPCC should not be construed to represent the complete incidence of national exposures to any substance.
